# Multi-omics profiling identifies berberine and salidroside as potential immunoregulatory compounds in radiation-induced skin injury

**DOI:** 10.3389/fimmu.2025.1666549

**Published:** 2025-09-18

**Authors:** Mengru Zhu, Yuxuan Shang, Mingjian Zhao, Jia Liu, Fengya Wang, Wei Zou, Bing Wang, Lukuan Liu, Jing Liu

**Affiliations:** ^1^ Stem Cell Clinical Research Center, The First Affiliated Hospital of Dalian Medical University, Dalian, Liaoning, China; ^2^ Department of Plastic Surgery, The First Affiliated Hospital of Dalian Medical University, Dalian, China; ^3^ Liaoning Key Laboratory of Frontier Technology of Stem Cell and Precision Medicine, Dalian Innovation Institute of Stem Cell and Precision Medicine, Dalian, China; ^4^ Department of Radiation Oncology, The First Affiliated Hospital of Dalian Medical University, Dalian, China

**Keywords:** radiation-induced skin injury, fibroblast, apoptosis, TGF-β, drug targets, immunotherapy

## Abstract

**Background:**

Radiation-induced skin injury is a significant concern in nuclear accidents and cancer radiotherapy (RT). Skin damage ranges from mild erythema to severe ulceration, which significantly affects the patients’ quality of life. While previous studies have highlighted the role of the apoptotic pathway, its precise mechanism in radiation-induced skin damage remains unclear.

**Method:**

First, single-cell RNA and RNA-seq datasets were searched using the Gene Expression Omnibus (GEO) database, and further subgroup analysis of fibroblast skin cells, pseudotime analysis, and cell-cell communication analysis were conducted to determine the important state during fibroblast differentiation. Next, we analyzed and screened the key genes in the RNA-seq data. Finally, we performed SA-β-gal staining, flow cytometry, and qRT-PCR in an *in vitro* irradiation model to validate TGFBR2 as a potential therapeutic target.

**Result:**

The apoptotic pathway plays a crucial role in fibroblasts. Subsequent analysis of fibroblast subtypes revealed different subtypes, including MMP3, Coch, Apod, and Eif4e. Enrichment analysis further demonstrated a significant upregulation of apoptosis in MMP3 fibroblasts (FIB). Pseudotime analysis indicated that MMP3 FIB exhibited high stemness within the fibroblast differentiation trajectory, while intercellular communication analysis underscored the critical role of TGF-β in fibroblast subtype interactions. Additionally, RNA-Seq analysis identified TGFBR2 as a key gene. *In vitro* experiments corroborated the essential function of TGFBR2 in radiation-induced skin injury. Lastly, molecular docking studies identified potential therapeutic agents, including Berberine and Salidroside.

**Conclusion:**

This study integrates single-cell and bulk transcriptomic analyses to reveal apoptosis-sensitive fibroblast subtypes involved in radiation-induced skin injury. MMP3^+^ fibroblasts were identified as a key apoptotic population with high stemness potential, and TGFBR2 was validated as a central regulatory target through molecular and cellular assays. Furthermore, Berberine and Salidroside were identified via molecular docking as potential compounds targeting TGFBR2. These findings provide mechanistic insight into fibroblast heterogeneity under radiation stress and offer a foundation for targeted therapeutic strategies.

## 1 Introduction

Radiation-induced skin injury is a serious issue in nuclear accidents and in radiotherapy for cancer. Radiation-induced skin injury occurs in approximately 95% of patients undergoing radiotherapy and ranges in severity from mild erythema to moist desquamation and ulceration. Some patients develop severe radiation dermatitis, which progresses to chronic radiation-induced skin injury, significantly affecting their quality of life (1–3). However, the molecular and cellular mechanisms underlying radiation-induced skin injury are not fully understood. It is currently believed that the DNA of skin cells, particularly keratinocytes and fibroblasts, is directly damaged by radiation, resulting in cell death and dysfunction accompanied by a series of abnormal oxidative stress and immune-inflammatory responses (4–6). Fibroblasts, as the primary damaged cells, can transform into persistent myofibroblasts after tissue injury, which is a key event in initiation and progression of fibrosis (4, 7–10). Therefore, further research on the mechanisms of action of fibroblasts in radiation-induced skin injury is of great importance to provide new targets and strategies for the treatment of radiation-induced skin injury.

Apoptosis is a form of programmed cell death that is of great importance for maintaining tissue homeostasis and preventing abnormal cell proliferation (11–13). During radiation-induced skin injury, many cells undergo apoptosis. Fibroblasts, the primary cell type susceptible to apoptosis due to radiation injury, undergo alterations in both quantity and functionality. These changes in fibroblasts can influence the biological behavior of surrounding cells through the secretion of apoptosis-related factors. This process exacerbates the degree of injury or affects the repair (14–16). Therefore, this study focused on the specific role of apoptosis in fibroblasts during radiation-induced skin injury, including the activation of apoptotic signaling pathways, changes in the expression of apoptosis-related genes, and the relationship between apoptosis and different fibroblast subtypes. Thus, we aimed to elucidate the potential regulatory mechanisms of apoptosis in the repair of radiation-induced skin injury.

With the rapid development of high-throughput sequencing technology and bioinformatics algorithms, advanced techniques, such as single-cell data analysis, transcriptome data analysis, and molecular docking, have been widely applied in biomedical research. In the study of radiation-induced skin injury and fibroblast apoptosis, these techniques provide powerful support for the in-depth analysis of cellular heterogeneity, gene expression regulatory networks, and drug target discovery. Single-cell data analysis can reveal heterogeneous changes in fibroblasts during radiation-induced skin injuries. Transcriptome data analysis enables the comprehensive elucidation of gene expression profile changes in fibroblasts during apoptosis. Molecular docking aids in predicting and validating potential drug targets and their interactions with proteins involved in fibroblast apoptosis. Therefore, this study focuses on the current application status and development trends of these techniques in the research of radiation-induced skin injury and fibroblast apoptosis, aiming to provide new ideas and methods for future research.

Transforming growth factor-beta receptor II (TGFBR2), a key mediator in the TGF-β signaling pathway, plays a central role not only in fibrogenesis but also in regulating immune homeostasis and immune evasion in various pathological contexts (17). Beyond its well-characterized role in fibroblast activation and extracellular matrix production, TGFBR2 signaling modulates immune responses by suppressing cytotoxic T cell activity, promoting regulatory T cell (Treg) differentiation, and dampening antigen presentation by dendritic cells (18). In the tumor microenvironment, persistent activation of TGFBR2 contributes to immune suppression and resistance to immunotherapy. Recent preclinical advances have highlighted TGFBR2 as a promising dual-function target that governs both fibrotic remodeling and immune modulation, particularly in settings such as cancer, chronic inflammation, and radiation-induced skin injury (19). Thus, a deeper mechanistic understanding of TGFBR2 in immune-fibrotic crosstalk is essential for the development of more precise and effective therapeutic strategies.

This study is the first to use multi-species data to explore TGFBR2 expression in fibroblasts from patients with post-radiation skin injury. The results suggest that TGFBR2 is a key gene in post-radiation skin injury, increasing the likelihood of our findings.

## 2 Materials and methods

### 2.1 Data collection and processing

Single-cell RNA data (GSE201447, GSE193564, and GSE193807) from mice, rats, and a nuclear accident patient with radiation-induced skin injury were downloaded from the Gene Expression Omnibus (GEO) database (20, 21). Data from either irradiated (γ-irradiation, 15 Gy) female mice 14 days post-treatment (n=4), or from age-matched controls (n=4). The rat scRNA data included five samples, and the irradiated skin (electron beam, 30 Gy) was collected on days 7, 14, 28, and 60 for RNA-seq. The skin of the control group was collected from four rats without radiation. Human irradiation scRNA data included two samples. Skin samples were obtained 160 days after irradiation from the right hand, which was exposed to an iridium-192 (192Ir) metal chain. Normal human skin tissue was obtained from the navel of the patient. Transcriptome data (GSE147733) were also obtained from the GEO database, including 7 normal samples and 13 radiation-induced skin fibroblast injury samples (22). In addition, the dataset GSE154559 was used as a validation dataset to verify the expression of key genes. GSE154599 contains 24 samples, 8 normal controls, and 16 fibroblasts from cancer patients receiving radiotherapy after surgery (23). The apoptosis genes were obtained from the GeneCard database, with a final selection of 182 apoptosis genes based on a relevance score greater than 10.

### 2.2 Single-cell RNA data analysis

The single-cell RNA data were analyzed using the “Seurat” package and visualization was performed by using “ggplot2,” “SCP,” “Nebulosa,” “scCustomize,” and “ggsci” R packages. Quality control retained cells with less than 5% mitochondrial genes, more than 200 genes, and genes expressed in at least 3 cells. The dataset was normalized using “LogNormalize’.” From each sample, 2,000 highly variable genes were selected for downstream analyses. Data integration and batch correction were performed using the “Harmony” R package (24, 25).

### 2.3 Cell-cell communication analysis

The “CellChat” R package was used to explore potential differences in cell–cell interactions between experimental and control skin samples following the official workflow. This tool simulates cell-cell communication by evaluating ligand-receptor pairs and their respective cofactors. Inferences regarding interactions between two cell types are based on the expression of receptors in one type and ligands in the other (26).

### 2.4 Functional enrichment analysis

The “irGSEA” package was used to score the two groups using the “UCell” and “AUCell” enrichment methods, respectively. A heatmap of the differentially enriched pathways between the two groups was generated. Subsequently, the “PercentageFeatureSet” function was used to import apoptosis-related genes and obtain the percentage of apoptosis-related genes in each cell (27–29).

### 2.5 Screening of core cells and functional enrichment analysis of their marker genes

To further clarify the role of fibroblasts in radiation-induced skin damage, the “FindAllMarkers” function in the “Seurat” package was utilized. Parameters were set as min.pct = 0.2 and only.pos = TRUE to identify marker genes for each cluster. The “MAST” algorithm was employed to detect differentially expressed genes (DEGs) during the process of selecting marker genes. The R package “clusterProfiler” was used to enrich the marker genes of core cells, achieving GO and KEGG functional annotations (30).

### 2.6 Pseudotime analysis

Cell stemness was assessed using the “CytoTRACE” R package to determine the temporal order of cell differentiation. The “Monocle” R package was used to analyze the pseudotime trajectory of tumor cells. Dimensionality reduction was achieved using the UMAP method, followed by the sorting of different cell subpopulations based on their pseudotime order. Pseudotime heatmaps were used to identify and represent genes that exhibit synchronous changes along the pseudotime trajectory (31, 32).

### 2.7 Machine learning for key gene selection

To further investigate gene interactions and functions, the STRING database was used to analyze and visualize gene interactions. The machine learning algorithm, the least absolute shrinkage and selection operator (LASSO), was constructed using the “caret” R package.

### 2.8 Cell culture and irradiation

HSF cells (human skin fibroblast used in *in vitro* experiments) were procured from the American Type Culture Collection (ATCC) and cultured in Dulbecco’s Modified Eagle Medium (DMEM; Thermo Fisher Scientific), which was supplemented with 10% fetal bovine serum (FBS) and 1% penicillin-streptomycin-amphotericin B suspension (P/S/AB) for the primary culture, all reagents were from Thermo Fisher Scientific. The culture medium was refreshed every day. The irradiation (IR) was performed using X-rays emitted by a Linear Accelerator (Elekta, Sweden, 6 MeV X-ray, 2 mm aluminum filter; 100 cm source-to-surface distance) at a dose of 16 Gy, with a dose rate of 6 Gy/min.For the IR group, HSF were plated in 6-well plates (2 × 10^5 cells/well) and maintained for 24 h before irradiation.

### 2.9 Senescence-associated β-galactosidase staining

HSF from both the control and IR groups were cultured for three days following radiation exposure. Subsequently, the cells were rinsed with PBS, fixed in fixative solution at room temperature for 15 min, and washed three times with PBS. The cells were then incubated overnight at 37 °C in freshly prepared β-galactosidase Staining Solution (Beyotime, Shanghai, China). Imaging was performed using an optical microscope (Olympus, Tokyo, Japan) with senescent cells exhibiting blue staining in the cytoplasm.

### 2.10 Quantitative real-time polymerase chain reaction

Total RNA was extracted using TRIzol reagent (Invitrogen), according to the manufacturer’s instructions. Optical density was used to determine the concentration and purity of RNA. An M-MLV Reverse Transcriptase Kit (Accurate Biology, Changsha, China) was used to synthesize cDNA. SYBR Green (Accurate Biology, Changsha, China) was used to perform Real-time PCR amplification, and the relative mRNA expression was calculated using the 2-ΔΔCt method and normalized to β-Actin expression. The study included n=3. Data are presented as the mean ± SD by paired two-tailed Student’s t-test, ***p < 0.001.

TGFBR2 Human qPCR Primer Pair show as follows:

Forward Sequence: GTCTGTGGATGACCTGGCTAAC.

Reverse Sequence: GACATCGGTCTGCTTGAAGGAC.

### 2.11 Apoptosis analysis

HSF were cultured in 6-well plates at a density of 2 × 10^5^ cells/well for 24h. Cells in the IR group were irradiated with X-rays (16 Gy, 6 GY/min). 48h after the irradiation, cells were trypsinized without EDTA and stained according to the instructions of the FITC Annexin V Apoptosis Detection Kit (Beyotime, Shanghai, China). Data were acquired using a BD FACS verse flow cytometer (BD Biosciences, NJ, USA) and analyzed using the FlowJo software. The study included n= 3. Data are presented as the mean ± SD by paired two-tailed Student’s t-test, **p < 0.01.

### 2.12 Molecular docking

Based on literature review, TGFBR2 was selected as the receptor protein, and “Berberine” and “Salidroside” were chosen as ligand small molecules. The receptor protein was imported into PyMOL 2.6.0 for dehydration. AutoDockTools 1.5.7 was used to connect, hydrogenate, and non-polarize the hydrogens, and then convert the protein to pdbqt format. The Grid function in AutoDockTools 1.5.7 was used to identify the binding regions of the protein receptor, that is, the active site. Small ligand molecules in mol2 format were imported into AutoDockTools for hydrogenation and charge calculation settings. Based on the docking pocket obtained from the previous step and the processed receptor and ligand, molecular docking was performed using AutoDock software, and the affinity and hydrogen bonds of amino acid residues were analyzed using PyMOL.

### 2.13 Molecular dynamic simulation

Gromacs2022.3 software was used for analysis, and added GAFF force field to small molecules through AmberTools22. Gaussian 16 W was used to hydrogenate small molecules and calculate their RESP potential. Static temperature was set of 300K and atmospheric pressure was set of 1 bar. Amber99sb-ildn was chosen as the force field, and water molecules (Tip3p water model) were selected as the solvent. The total charge of the simulation system was neutralized by adding an appropriate amount of Na+ ions. The energy minimization was carried out by using the steepest descent method, and then 100,000 steps of isothermal and isobaric ensemble (NVT) equilibrium and isothermal and isobaric ensemble (NPT) equilibrium were performed respectively. The coupling constant was set to 0.1 ps, and the whole process lasted for 100 ps. Finally, the free molecular dynamics simulation consisted of 5000000 steps, with a step size of 2fs and a total duration of 100ns. After the simulation was completed, the trajectories were analyzed using built-in software tools, and the protein rotation radius, root mean square fluctuation (RMSF), and root mean square deviation (RMSD) of each amino acid trajectory were calculated based on data such as free energy (MMGBSA) and free energy morphology.

## 3 Results

**Figure 1:**
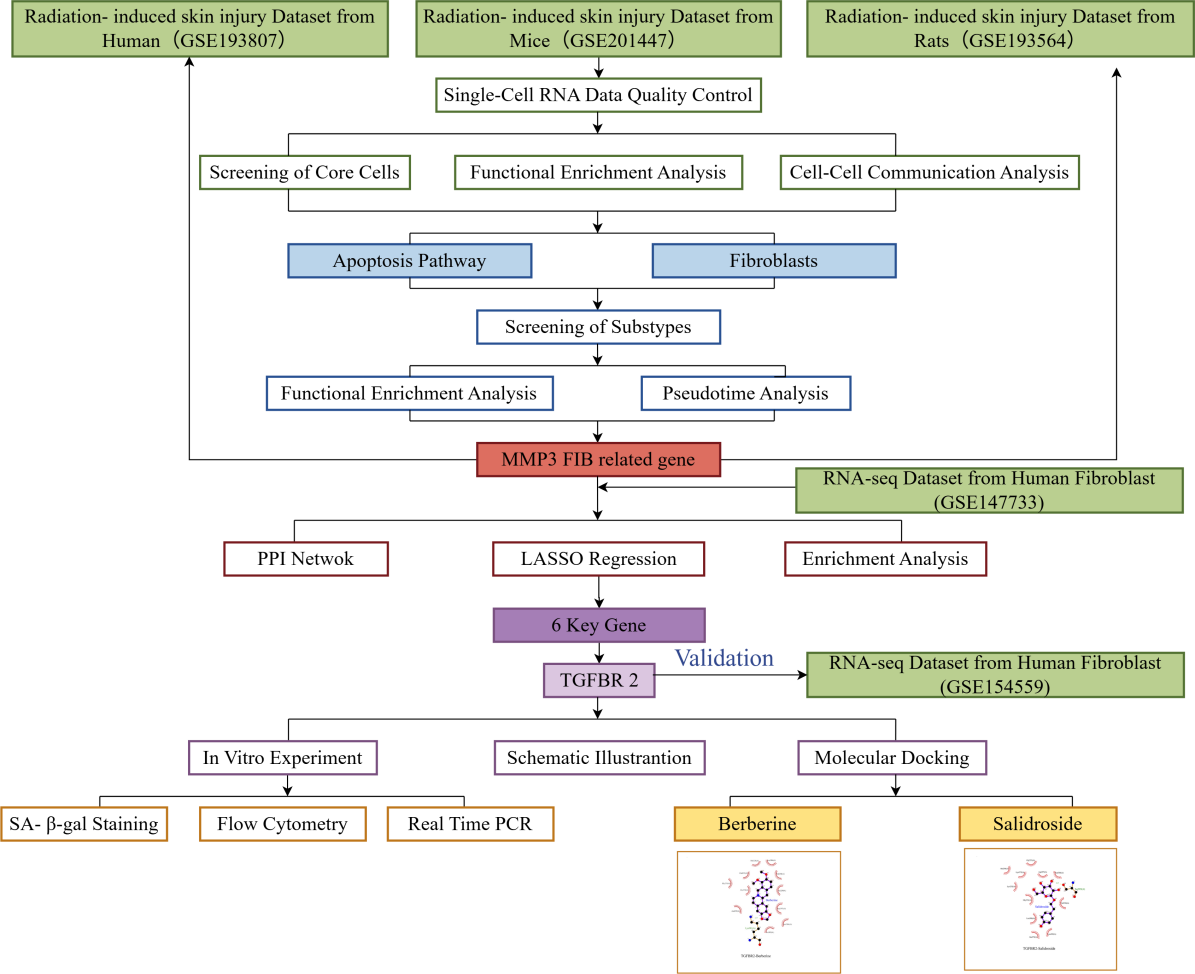
Flow chart of this study (Figdraw, ID:UPSYS00f00).

The flow chart of this study is detailed in **Figure 1**.

### 3.1 Annotation result of single-cell data

**Figure 2:**
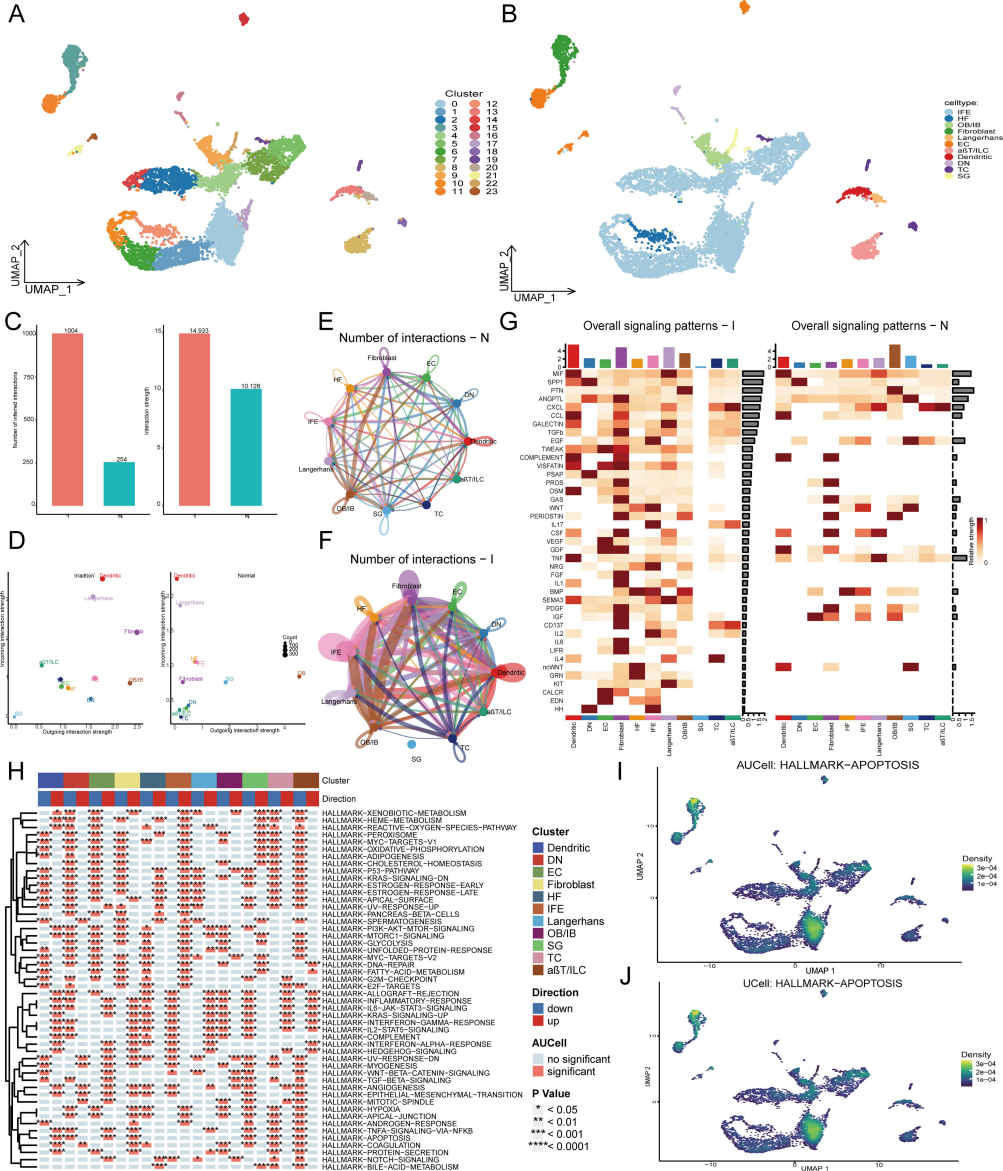
Single cell RNA analysis of radiation-induced skin injury. **(A)** All cells in 8 samples were clustered into 24 clusters. **(B)** After cell annotation, 24 cell clusters were annotated as 11 types of cells. **(C)** The cell communication analysis shows the number and strength of interaction between the control group and the irradiation group. **(D)**The scatter plot shows the correlation between input and output interaction strength of different cells in the irradiation group and the control group. **(E, F)** Network diagrams visualize the number of cell communications in the control group and the irradiation group. **(G)** Heatmaps show the involvement of different types of cells in signaling pathways in the normal group and the irradiation group. **(H)** irGSEA enrichment analysis showed enrichment of pathways among different cells. **(I, J)** AUCell and UCell algorithm calculated Apoptosis-enriched regions and Apoptosis related gene expression.

At the beginning, quality control was applied to the single-cell sequencing dataset by setting the threshold as described above and using the “Harmony” R package to remove batch effects caused by different samples. Dimensionality reduction was achieved through the Uniform Manifold Approximation and Projection (UMAP) method, whereas clustering was conducted using the K-Nearest Neighbors (KNN) algorithm with a resolution parameter set at 0.8. All cells included in the study were divided into 24 different clusters through dimensionality reduction clustering analysis, and cell annotations were performed based on the expression of characteristic genes within each cluster and related article, resulting in a total of 11 cell types (20). (**Figures 2A, B**, **Supplementary Figures 1A, B**).

### 3.2 Cell communication and enrichment analysis

This study analyzed cell communication using various visualizations. A bar graph (**Figure 2C**) shows increased communication intensity and quantity in the irradiation group compared to the control group. Scatter plots (**Figure 2D**) highlight the strong input and output strengths of fibroblasts across cell types. A network map (**Figures 2E, F**) illustrates the diversity of intercellular signal interactions between groups. Heatmaps (**Figure 2G**, **Supplementary Figure 1D**) demonstrated that fibroblasts play a significant role in information flow, particularly in the TGF-β signaling pathway, which is crucial for radiation-induced skin injury, where fibroblasts predominantly act as signal receivers.

### 3.3 Enrichment analysis of different cell types in single cells data

To further investigate the expression of apoptosis pathways across different cell types, enrichment analysis utilizing the “irGSEA” R package revealed pronounced expression of apoptosis pathways in fibroblasts (**Figure 2H**). The expression levels of apoptosis pathways were quantified using the “UCell” and “AUCell” algorithms, demonstrating a significant overexpression of these pathways in fibroblasts (**Figures 2I, J**). Lastly, the “PercentageFeatureSet” function was employed to incorporate apoptosis-related genes, thereby calculating the proportion of apoptosis genes within each cell. The cells were subsequently categorized into low- and high-apoptosis groups. (**Supplementary Figure 1C**).

### 3.4 Subtype analysis of fibroblasts

**Figure 3:**
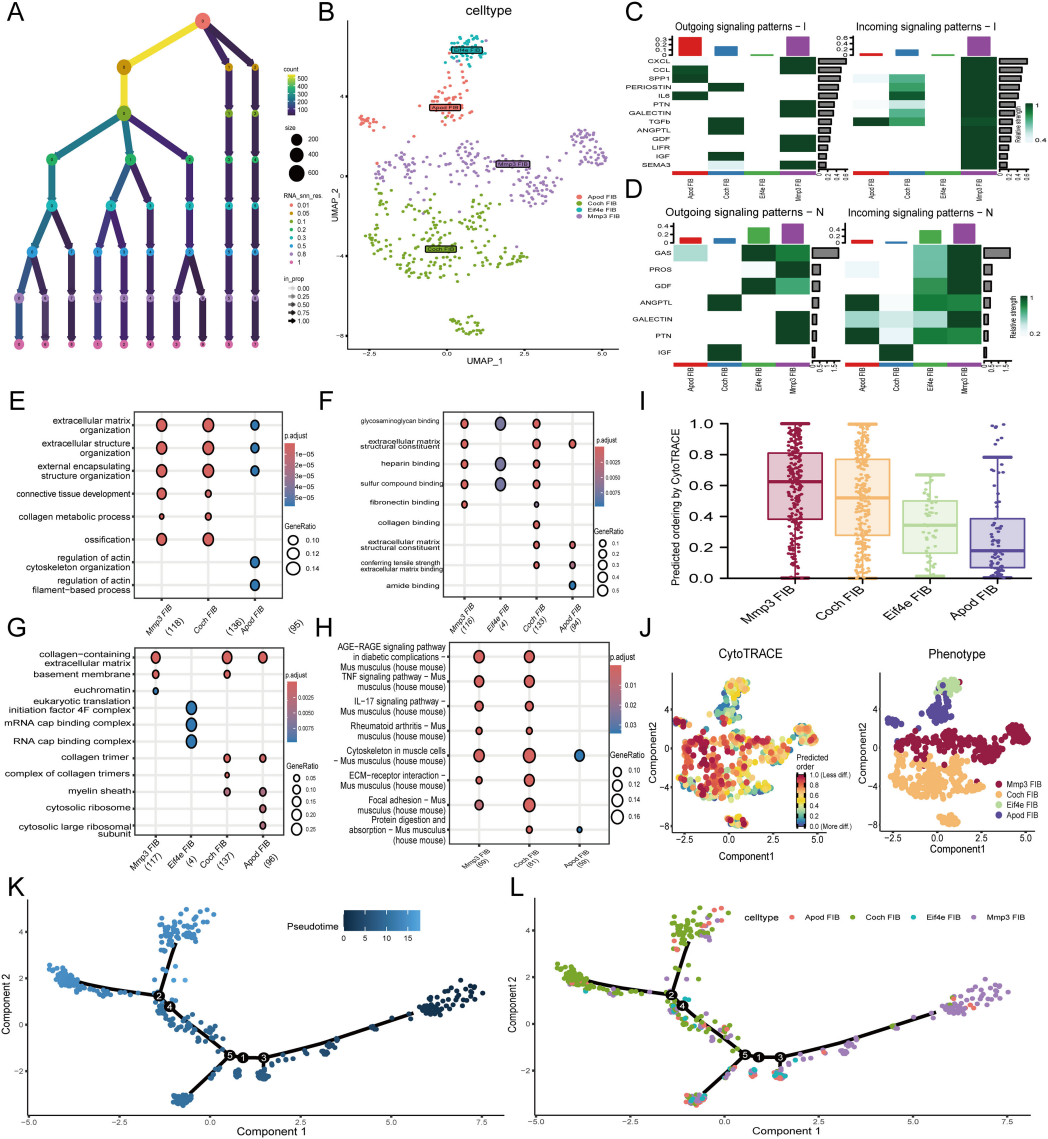
Single-cell RNA analysis of fibroblast subtypes. **(A)** Dendrogram visualizing different thresholds. **(B)** Annotation results of fibroblast subtypes. **(C, D)** The heatmaps show the interactions between irradiated and control groups of fibroblast subtypes. Enrichment analysis results of GO pathway enrichment analysis among different fibroblast subtypes **(E)** BP **(F)** CC **(G)** MF. **(H)** Results of KEGG pathway among different fibroblast subtypes. **(I)** Barplot demonstrated the predicted ordering by CytoTRACE of fibroblast subtypes. **(J)** The left figure represented the analysis of the differentiation of fibroblast subtypes using CytoTRACE. The color could represent the level of differentiation. The right figure represented the CytoTRACE results displayed according to different fibroblast subtypes. **(K)** UMAP plot demonstrated the differentiation of fibroblast on the pseudotime trajectory. **(L)** UMAP plot demonstrated fibroblast subtypes distribution on the pseudotime trajectory. BP, Biological Process; CC, Cellular Component; MF, Molecular Function.

To elucidate the mechanisms of apoptosis in fibroblasts, we focused on fibroblasts and conducted detailed analyses. Fibroblast cells were isolated and subjected to repeated dimensionality reduction and clustering analyses. We also present the clustering threshold selection results as a decision tree (**Figures 3A**). We successfully identified and annotated five distinct cell subtypes (**Supplementary Figure 2A**). The expression of the marker genes in these cells is shown in the bubble plot, where none of the Col1a1 and Col3a1 genes were expressed in cluster 4 cells; Therefore, cluster 4 cells were rounded off (**Supplementary Figure 2B**). We annotated the remaining four cell subtypes as Apod FIB (fibroblast identified in single-cell RNA sequencing data), Coch FIB, Eif4e FIB, and Mmp3 FIB (**Figure 3B**).

### 3.5 Interactions among fibroblast subtypes

In the radiation-induced skin injury group, 44 cell communication pathways were identified in the radiation-induced skin injury group, whereas 26 pathways were found in the control group (**Supplementary Figure 2C**). Cell communication among fibroblasts after radiation-induced skin injury significantly increased, with MMP3 being highly connected (**Supplementary Figure 2D**). Cell communication analysis showed that the MMP3 FIB was the primary signal sender and receiver among all fibroblasts (**Supplementary Figure 2E**). Specifically, compared to the control group, MMP3 FIB in the radiation skin injury group communicated more frequently and was involved in more pathways related to post-radiation biological processes, including TGF-β, SPP1, CXCL, and particularly the TGF-β signaling pathway, which plays a crucial role in radiation-induced skin damage (**Figures 3C, D**).

### 3.6 Enrichment analysis of fibroblast subtype

To further clarify the roles of the different fibroblast subtypes in radiation-induced skin injury, GO and KEGG enrichment analyses were performed. MMP3 FIB was enriched in pathways such as AGE-RAGE signaling in diabetic complications, TNF signaling pathway, IL-17 signaling pathway, and ECM-receptor interaction, as well as in cellular functions such as extracellular matrix organization, collagen-containing extracellular matrix, and glycosaminoglycan binding. It means that it plays an important role in the repair process (**Figures 3E-H**). Furthermore, KEGG analysis of enriched pathways in different cell subtypes showed that in addition to high expression in the apoptosis pathway, MMP3 FIB cells were significantly upregulated in pathways such as INFLAMMATORY-RESPONSE, PI3K-AKT-MTOR-SIGNALING, and IL6-JAK-STAT3-SIGNALING, also indicating the important role of MMP3 FIB in radiation-induced skin injury (**Supplementary Figure 2F**).

### 3.7 Pseudotime analysis of fibroblast subtypes

The “CytoTRACE” R package was used to analyze the characteristics of fibroblast subtype differentiation. The results showed that MMP3 FIB cells had the highest stemness compared to the other subtypes (**Figures 3I, J**). R package “Monocle” was used to analyze fibroblast subtype differentiation. Compared with other fibroblast subtypes, MMP3 had the lowest degree of differentiation, consistent with the CytoTRACE results. Based on this, we examined the trajectories of pseudotime, different cell types, and the cell cycle (**Figures 3K, L**, **Supplementary Figures 2G, H**). As pseudotime progressed, the changes in the expression of differentially expressed genes are shown (**Supplementary Figure 2I**).

### 3.8 Gene expression and key gene screening in MMP3 FIB

**Figure 4:**
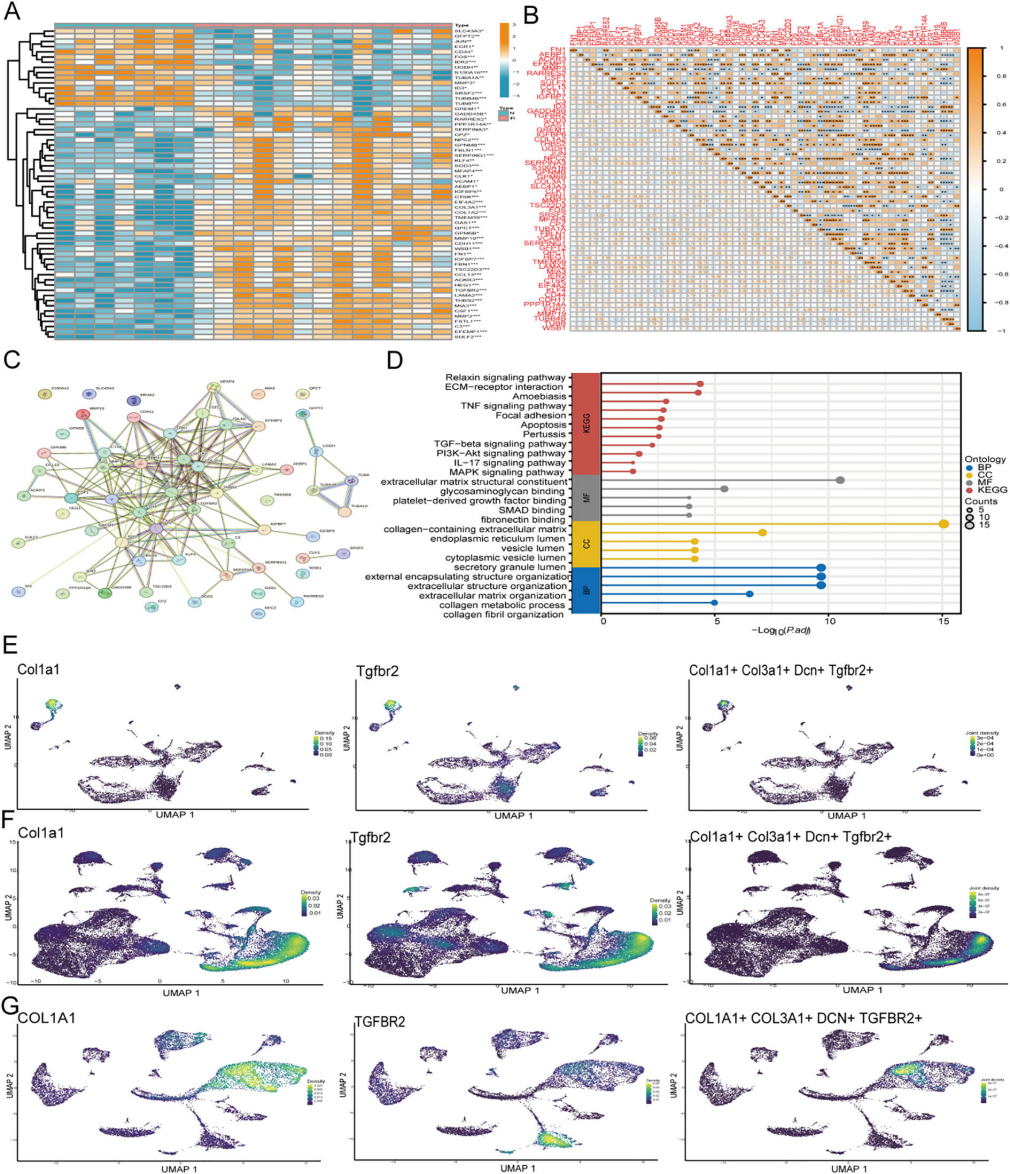
Gene expression and enrichment analysis of MMP3 FIB subtypes and validation. **(A)** Heatmap demonstrating the expression of MMP3 FIB isoform genes **(B)** Correlation between MMP3 FIB isoform genes. **(C)** String database presentation correlation between MMP3 FIB subtype genes **(D)** KEGG and GO function enrichment results of MMP3 FIB subtype gene. **(E-G)** Expression of TGFBR2 and fibroblast related genes in the mice, rat, and human single-cell data. FIB, fibroblast identified in single-cell RNA sequencing data.

From single-cell sequencing data, 105 DEGs were obtained for MMP3 FIB, with screening criteria set at logFC > 0.5 and adjPvalue < 0.05. Ultimately, 60 genes were differentially expressed (**Figures 4A**, **Supplementary Table 1**). The interactions among genes are shown, and to further determine gene interactions, the String database was used to analyze gene interactions (**Figures 4B, C**). The results showed that most genes were positively correlated, and the KEGG database indicated that genes were enriched in pathways such as extracellular matrix organization, apoptosis, and TGF-ß (**Figure 4D**). The LASSO machine learning algorithm was used to screen for key genes, and ultimately, six genes were selected: TUBB, S100A16, MMP19, SULF2, TMEM59, and TGFBR2 (**Supplementary Figure 2J**). Finally, to further verify the important role of TGFBR2 in radioactive skin injury, we validated it using single-cell data from rats and radioactive damage patients, and the results showed that the TGFBR2 gene was significantly clustered in fibroblasts (**Figures 4E-G**).

### 3.9 Radiation induces HSF senescence and apoptosis by upregulating TGFBR2

**Figure 5:**
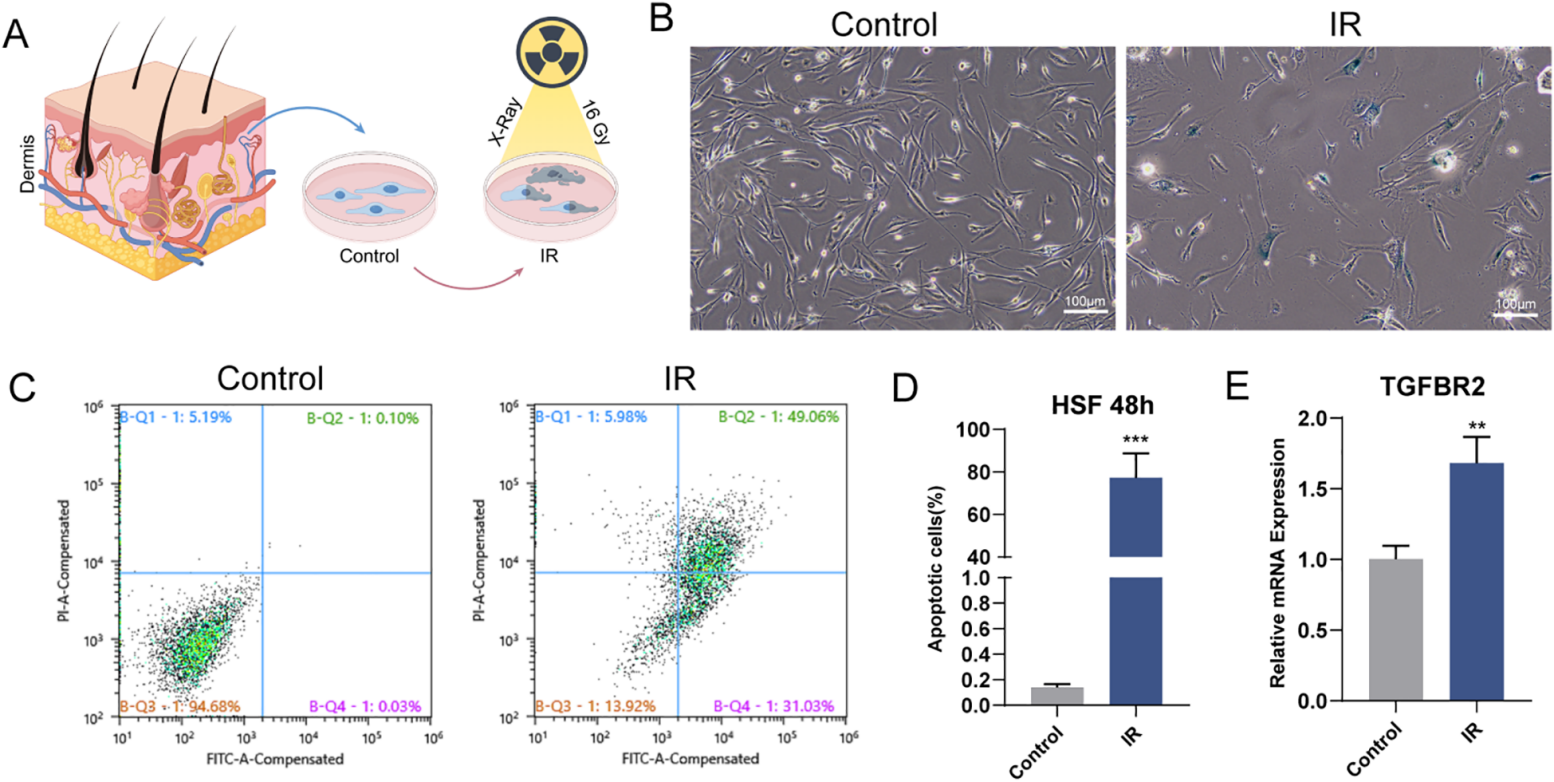
Radiation induces HSF senescence and apoptosis by upregulating TGFBR2 **(A)** Schematic diagram of the control group and experimental group. **(B)** Representative images of SA-β-gal staining in HSF (scale bar, 100 μm). **(C, D)** Flow cytometry to compare apoptosis ratio of IR with control group. n=3, ***p < 0.001. **(E)** Quantitation of mRNA levels of TGFBR2, n=3, **P < 0.01.

To determine whether TGFBR2 is a pivotal gene in fibroblasts affected by radiation, we developed an *in vitro* cellular radiation model, as depicted in the schematic diagram (**Figure 5A**). Radiation exposure led to an increase in SA-β-gal-positive cells in human skin fibroblasts (HSF) (**Figure 5B**). Flow cytometry analysis revealed that a single high dose of X-ray significantly induced apoptosis in HSF, encompassing both early- and late-stage apoptosis (**Figures 5C, D**). 48h post-irradiation, the mRNA expression of TGFBR2 was found to be significantly upregulated, as determined by RT-PCR assay, corroborating the results of key gene screening (**Figure 5E**).

### 3.10 Molecular docking

Traditional Chinese medicine (TCM) has been proven to be highly effective in treating radiation-induced skin injuries and is widely used for this purpose. Through a literature review, four herbal medicines (Berberine and Salidroside) were identified as potentially beneficial in the treatment of radiation-induced skin injuries. Molecular docking studies were conducted to explore whether these herbs regulated apoptosis by targeting TGFBR2.

**Figure 6:**
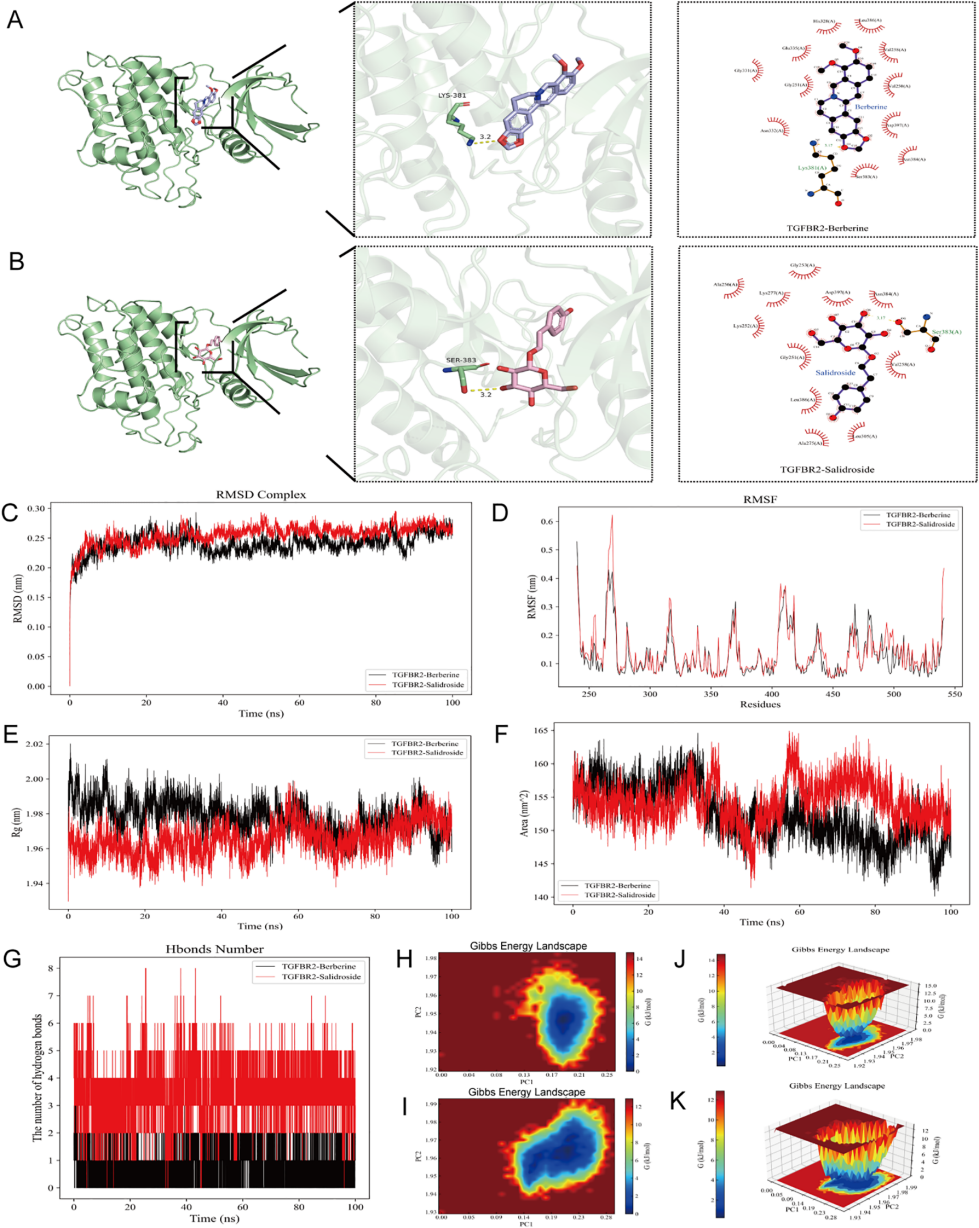
Molecular docking and molecular dynamic results. **(A)** TGFBR2 - Berberine molecular docking. **(B)** TGFBR2 -Salidroside molecular docking.the molecular dynamics simulation analyses of the TGFBR2-Berberine and TGFBR2-Salidroside complexes over a 100 ns simulation period, including **(C)** RMSD, **(D)** RMSF, **(E)** Rg, **(F)** SASA and **(G)** the number of hydrogen bonds. **(H, J)** 2D and 3D plot of Gibbs free energy analysis of TGFBR2 - Berberine complexes. **(I, K)** 2D and 3D plot of Gibbs free energy analysis of TGFBR2 -Salidroside complexes.

The results of molecular docking showed that each of the four active compounds had a binding energy with the TGFBR2 protein target lower than -5 kcal/mol, indicating spontaneous binding between the ligand and the receptor. The magnitude of the lowest binding energy was negatively correlated with the binding energy of small molecules to the target protein. Intermolecular interactions, such as hydrogen bonds, aromatic interactions, and other types of interactions, can lead to tight binding, further suggesting that the active components and target protein can form more stable conformations, thus enhancing their binding affinity. For each docking simulation, 50 formations were generated, and the conformation with the best binding energy was selected for visualization, as shown in **Figures 6A, B**. Therefore, TGFBR2 could be a potential target for the treatment of radiation-induced skin injuries using Berberine, and Salidroside.

### 3.11 Molecular dynamic simulation result

Molecular dynamic simulations were performed to verify the molecular docking simulation results of TGFBR2. The RMSD curves for the TGFBR2-Berberine and TGFBR2-Salidroside complexes showed equilibrium after 30 ns, with average RMSD values of 0.2 nm (**Figure 6C**). RMSF analysis identified key flexible regions in TGFBR2 proteins, specifically around amino acid residues 260–280 for TGFBR2-Berberine and TGFBR2-Salidroside (**Figure 6D**). The Rg values for the two complexes maintained equilibrium, with a means of 1.97 nm (**Figure 6E**). The corresponding solvent-accessible surface area (SASA) values for Berberine and Salidroside were stable at 155 nm² and 150 nm2 (**Figure 6F**). During the 100 ns simulation, the hydrogen bond numbers for the TGFBR2-Berberine and TGFBR2-Salidroside complexes ranged from to 0–3 and 0–8 respectively (**Figure 6G**). Gibbs free energy landscapes 2D and 3D plots were generated using the RMSD, Rg, and Gibbs free energy values of the complexes (**Figures 6H-K**). These maps provide insights into the stability of the complexes, with regions shaded in blue and darker blue indicating lower energy states achievable in steady-state conformations. In contrast, weak or unstable protein-ligand interactions yield multiple surface-rough minimum energy clusters, whereas strong interactions are represented by nearly single and smooth energy clusters in the potential energy distribution. Specifically, when Berberine and Salidroside are docked with the protein receptor, their corresponding PC1 values fall within the range of 0.0-0.25, while the PC2 value is stable in the range of 1.92-1.98. These values were combined with the RMSD curve of the complex to confirm its stability.

**Figure 7:**
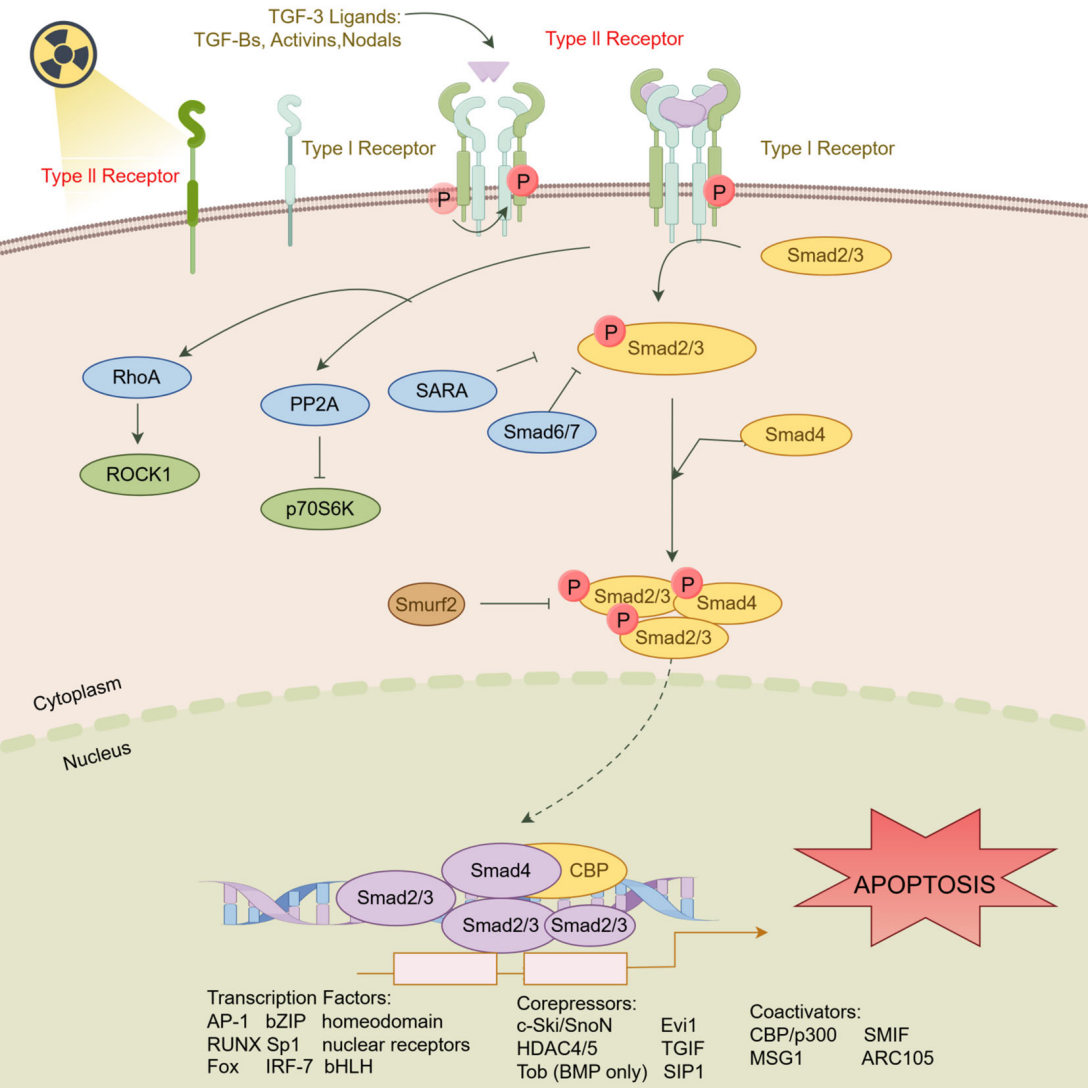
Schematic of the mechanism of TGFBR2 in mediating radiation induced HSF apoptosis. (Figdraw, ID: WPTRS15c5c).

The potential mechanism by which TGFBR2 mediates radiation-induced apoptosis in HSF is shown in the schematic (**Figure 7**).

## 4 Discussion

Radiation skin injury is a common skin injury after cancer radiation therapy, and about 95% of radiation therapy patients experience radiation skin injury, seriously affecting their quality of life (3, 33). Fibroblasts, as an important component of skin tissue, are crucial for maintaining skin homeostasis and promoting wound repair (34, 35). Apoptosis is a programmed cell death that plays a crucial role in maintaining tissue homeostasis and preventing abnormal cell proliferation (11–13). During radiation-induced skin damage, a large number of cells undergo apoptosis, among which fibroblasts are a type of cell that is prone to apoptosis due to radiation damage. These quantitative and functional modulations have been clinically associated with aggravated injury progression and compromised healing outcomes (14–16).Therefore, in-depth research on the key role of fibroblast apoptosis in radiation-induced skin injury is crucial for elucidating the mechanism of injury and developing new therapeutic strategies.

This study used single-cell data analysis and transcriptome data analysis to screen key genes and identify potential biological targets through molecular docking analysis. Subsequently, drug molecular dynamics simulation analysis was used to examine binding stability. Initially, this study used mouse single-cell data analysis to annotate cells by specifically expressing genes on the cell surface and ultimately obtained a total of 11 types of cells. Further analysis of intercellular communication and pathway enrichment showed that the signal intensity and quantity were high in fibroblasts, and the apoptotic pathway was significantly elevated. To further clarify the potential mechanisms involved, the regions with high expression of apoptosis pathways were analyzed using algorithms such as AUCell. The results showed that apoptosis-related genes and pathways are significantly overexpressed in the fibroblast region. Subsequently, to further clarify the potential mechanism of fibroblasts in the process of radiation-induced skin injury, an important fibroblast subtype named MMP3-FIB was identified through further classification of fibroblasts. The results of the pseudo-temporal analysis showed that MMP3-FIB was the first cell to distinguish between all fibroblast subtypes, serving as both a signal transmitter and a signal receiver for most cellular pathways. The results of enrichment analysis showed that MMP3-FIB cells were significantly enriched in various inflammatory response pathways, indicating that MMP3 cells play a crucial role in radiation-induced skin injury. In addition, the results of GSEA suggest that the apoptotic pathway in MMP3-FIB is significantly upregulated compared to other fibroblast subtypes, further highlighting the important role of the apoptotic pathway in MMP3-FIB cells. To further elucidate the mechanism of action of MMP3-FIB, transcriptome analysis was conducted to verify the differential expression of genes in MMP3-FIB. In addition, the LASSO machine learning algorithm is used to identify feature genes. Subsequently, six characteristic genes were identified, namely TUBB, S100A16, MMP19, SULF2, TMEM59, and TGFBR2. The TGFBR2 gene, also known as transforming growth factor beta receptor type II (TGF-ß receptor II), plays a crucial role in fibroblasts. The TGF-ß signaling pathway is an important signaling pathway in radiation-induced skin injury, which participates in the repair process of radiation-induced skin injury by promoting inflammatory response, tissue repair, and fibrosis processes. Mmp3 cells are the main signal receptors of the TGF-ß signaling pathway (17, 36–38) Given that TGFBR2 is the main receptor in the TGF-ß signaling pathway, its role in radiation-induced skin injury cannot be ignored (39). We successfully constructed an acute radiation-induced injury model of fibroblasts by using X-rays emitted by a linear accelerator. Through *in vitro* validation, it has been demonstrated that the TGFBR2 gene is significantly upregulated after radiation and is a key target of injury. To further provide more selectivity for clinical treatment, potential therapeutic drugs were identified through molecular docking, including berberine and salidroside, both of which have good binding energies with TGFBR2. Subsequent molecular dynamics simulations validated this result, providing new possibilities for the mechanism of these drugs in treating radiation-induced skin injury. At the same time, validation analysis was conducted using single-cell samples of radiation-induced skin injury in rats and humans, which confirmed the significant upregulation of TGFBR2 expression in irradiated tissues, mainly in the fibroblast region. To our knowledge, this is the first time that multi-species single-cell data has been used for analysis.

In the context of radiation-induced skin injury, the inflammatory response is a critical early pathological process (10, 40). The TGF -ß signaling pathway may affect this process by regulating the expression of inflammatory factors and promoting the infiltration of inflammatory cells (41). Long-term or high-dose radiation exposure may lead to fibrosis formation, and the activation of TGFBR2 is a key step in this process. Current research has confirmed that the SMAD pathway is the main typical pathway of the TGF - ß signaling pathway, which is recognized by TGFBR2 (41, 42). Previous studies have demonstrated that downstream factors of SMAD signaling, particularly Smad2/Smad3, are considered key mediators of TGF-ß signaling in tissue fibrosis (43). Moreover, fibroblast-to-myofibroblast transition represents a hallmark of fibrosis, whereas fibroblasts may instead undergo apoptosis during acute injury phases (44). The dual role of TGFBR2—facilitating tissue repair while also driving pathological fibrosis—warrants functional validation (45). To verify the functional involvement of TGFBR2 in radiation-induced skin injury, we established an *in vitro* X-ray irradiation model using HSF. X-ray exposure led to a significant increase in SA-β-gal-positive cells, indicating early senescence, and induced apoptosis at both early and late stages, as shown by flow cytometry. Importantly, qRT-PCR analysis showed a marked upregulation of TGFBR2 mRNA expression at 48 hours post-irradiation, aligning with our transcriptomic screening results. These findings support that radiation induces fibroblast senescence and apoptosis, at least in part, through TGFBR2-mediated signaling. Therefore, TGFBR2 may serve as a critical regulatory node linking acute fibroblast apoptosis and chronic fibrotic remodeling in radiation-damaged skin.

In the molecular docking analysis, both Berberine and Salidroside demonstrated strong binding affinities to TGFBR2, suggesting their potential as therapeutic agents capable of modulating this critical receptor involved in radiation-induced skin injury. Berberine exhibited stable interaction through multiple hydrogen bonds and hydrophobic contacts within the active pocket of TGFBR2, while Salidroside also established a favorable binding conformation with consistent hydrogen bonding. These interactions were further validated by molecular dynamics simulations, which confirmed the structural stability of the compound–receptor complexes over time. Berberine, exhibits dual activity by inhibiting fibroblast activation and modulating immune checkpoints. It downregulates IFN-γ–induced PD-L1 expression via the ITGB1/FAK axis, thereby enhancing T cell–mediated cytotoxicity and mitigating immune suppression (46). Recent evidence has further demonstrated that berberine enhances antitumor immunity by remodeling the immune microenvironment. In colorectal cancer models, it increases CD8^+^ T cell infiltration and cytotoxicity while reducing the abundance of regulatory T cells (Tregs) and myeloid-derived suppressor cells (MDSCs), thus reversing immunosuppressive conditions (47). Salidroside, a major active compound in traditional Chinese medicine, has shown promise in modulating immune responses and fibrosis, particularly through regulation of the TGF-β signaling pathway. In addition, Salidroside-loaded liposomal formulations have been reported to enhance dendritic cells activation and immune responses (48). Specifically, these nanocarriers promote dendritic cells maturation and significantly improve their antigen presentation and lymphocyte proliferation capacity in mixed lymphocyte reactions, thereby inducing robust cellular and humoral immune responses.

Taken together, these findings support that both Berberine and Salidroside may exert therapeutic effects not only by targeting fibroblast-mediated injury pathways but also by modulating the immune microenvironment associated with radiation-induced skin injury. Their ability to regulate key immune components—such as T cell activity, macrophage polarization, and cytokine production—provides compelling evidence of their dual action. This dual functionality positions these natural compounds as promising agents in precision immunomodulatory strategies, offering new perspectives for integrative treatment approaches in radiation-induced skin injury.

Despite the promising immunomodulatory potential of Berberine and Salidroside identified in this study, their inherent physicochemical limitations—including poor water solubility, low oral bioavailability, rapid metabolism, and limited tissue targeting—pose significant challenges to the clinical translation. Recent advances in nanodelivery technologies have demonstrated promising solutions. Liposomal and nanoemulsion systems have been shown to significantly enhance the oral bioavailability and intestinal permeability of Berberine (49, 50), while nanocrystals and transfersomes improved transdermal delivery by enhancing skin penetration (51, 52). For Salidroside, nanostructured lipid carriers exhibited improved stability and absorption (53). Collectively, future studies should prioritize the development and optimization of these delivery systems to enhance their pharmacokinetics, targeted accumulation, and therapeutic outcomes, particularly in the context of inflammatory and radiation-induced skin injury.

This study also has some limitations. Firstly, although we identified the key subtype MMP3-FIB and the key gene TGFBR2 through single-cell data analysis, and validated their expression *in vitro* experiments, further experiments are needed in the future to explore the specific functional roles of TGFBR2 and TGF-ß related pathways in the pathogenesis and treatment of radiation-induced skin injury. Secondly, the findings of this study are mainly based on public databases and lack detailed clinical validation. Although drug docking shows the possibility of new drugs, future studies should explore their potential synergistic effects when combined with immunotherapeutic agents or nanocarrier-based delivery systems to enhance specificity and efficacy. Therefore, large-scale clinical data will help further explore the correlation between these signature genes and diverse clinical outcomes.

## 5 Conclusion

Fibroblasts play a vital role in maintaining skin homeostasis and orchestrating the repair response following the radiation. In this study, we identified the MMP3^+^ fibroblast subtype as a key apoptosis-related population, with TGFBR2 emerging as a central regulatory gene. Through integrated transcriptomic analysis and machine learning, TGFBR2 was validated as a potential therapeutic target, and candidate compounds such as Berberine and Salidroside were identified via molecular docking. These compounds not only target fibroblast-driven fibrotic pathways but also exhibit immunomodulatory potential, offering dual-action therapeutic value. Our findings highlight the functional heterogeneity of fibroblasts in radiation-induced skin injury and propose a precision strategy that integrates natural product-based targeting and immune modulation. Further investigation into fibroblast–immune cell interactions and targeted delivery approaches may provide new avenues for personalized treatment and regenerative medicine in radiation-induced skin injury.

## Data Availability

The original contributions presented in the study are included in the article/**Supplementary Material**. Further inquiries can be directed to the corresponding authors.

## Supplementary material

Supplementary Figure 1
**(A)** The cells were annotated according to the surface marker genes of different cell types. **(B)** Fibroblast surface marker gene expression in the clusters. **(C)**Apoptosis-related gene expression. **(D)** Incoming signaling pathways in each cell type. **(E)** Outgoing signaling pathways in each cell type

Supplementary Figure 2
**(A)** The UMAP map of fibroblasts at K = 0.2. **(B)** Characteristically expressed genes in different fibroblast subpopulations. **(C)** The bar plot shows the number and intensity of fibroblast signaling pathways in the irradiation and normal groups. **(D)** Scatter plot of the intensity of cell signaling communication of fibroblast subtypes in the irradiated and normal groups. **(E)** Outgoing and incoming signaling pathways of different fibroblast subtypes. **(F)** Heatmap showing the results of AUCell enrichment analysis of fibroblast subtypes. **(G)** UMAP plot showing the cell cycle of fibroblasts in the pseudotime trajectory. **(H)** UMAP plot showing a cluster of fibroblasts in the pseudotime trajectory. **(I)** Fibroblast differentiation differential gene heatmap. **(J)** Lasso machine-learning algorithm threshold selection. **(K)** The bar plot shows TGFBR2 gene expression in the irradiated and normal groups.

Supplementary Table 1 MMP3 fibroblast subtype related genes(top50).
