# Supplementary material for: Multi-omics profiling identifies berberine and salidroside as potential immunoregulatory compounds in radiation-induced skin injury
Source: Front Immunol. 2025 Sep 18;16:1666549. doi: 10.3389/fimmu.2025.1666549 (PMC12488636; doi:10.3389/fimmu.2025.1666549)
Supplement: Supplementary Figure 1 — (A) The cells were annotated according to the surface marker genes of different cell types. (B) Fibroblast surface marker gene expression in the clusters. (C)Apoptosis-related gene expression. (D) Incoming signaling pathways in each cell type. (E) Outgoing signaling pathways in each cell type [file Supplementaryfile1.docx]

Supplymentary Materials

Multi-Omics Profiling Identifies Berberine and Salidroside as Potential Immunoregulatory Compounds in Radiation-Induced Skin Injury


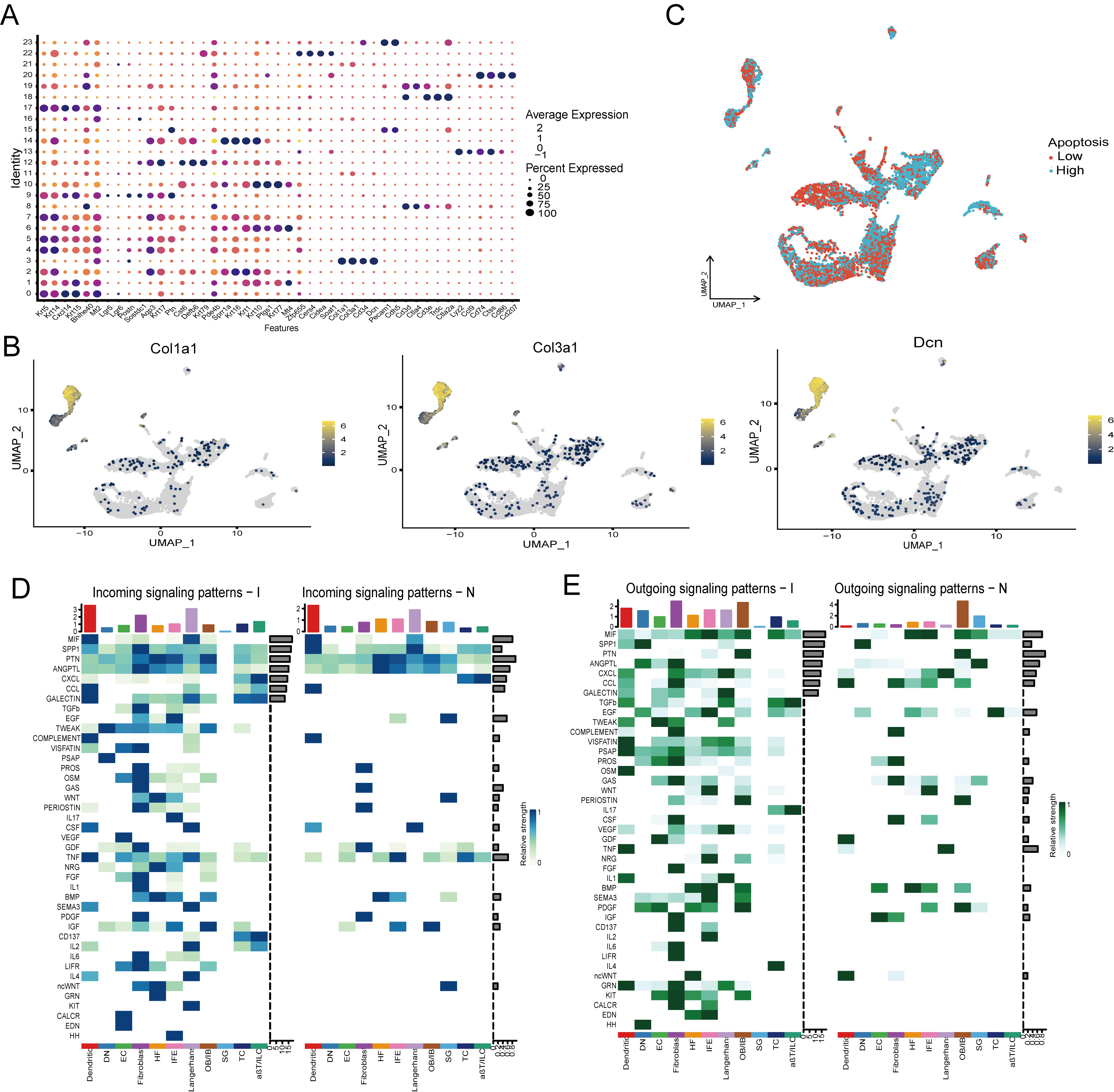


Supplementary Figure1. (A) The cells were annotated according to the surface marker genes of different cell types. (B) Fibroblast surface marker gene expression in the clusters. (C)Apoptosis-related gene expression. (D) Incoming signaling pathways in each cell type. (E) Outgoing signaling pathways in each cell type.


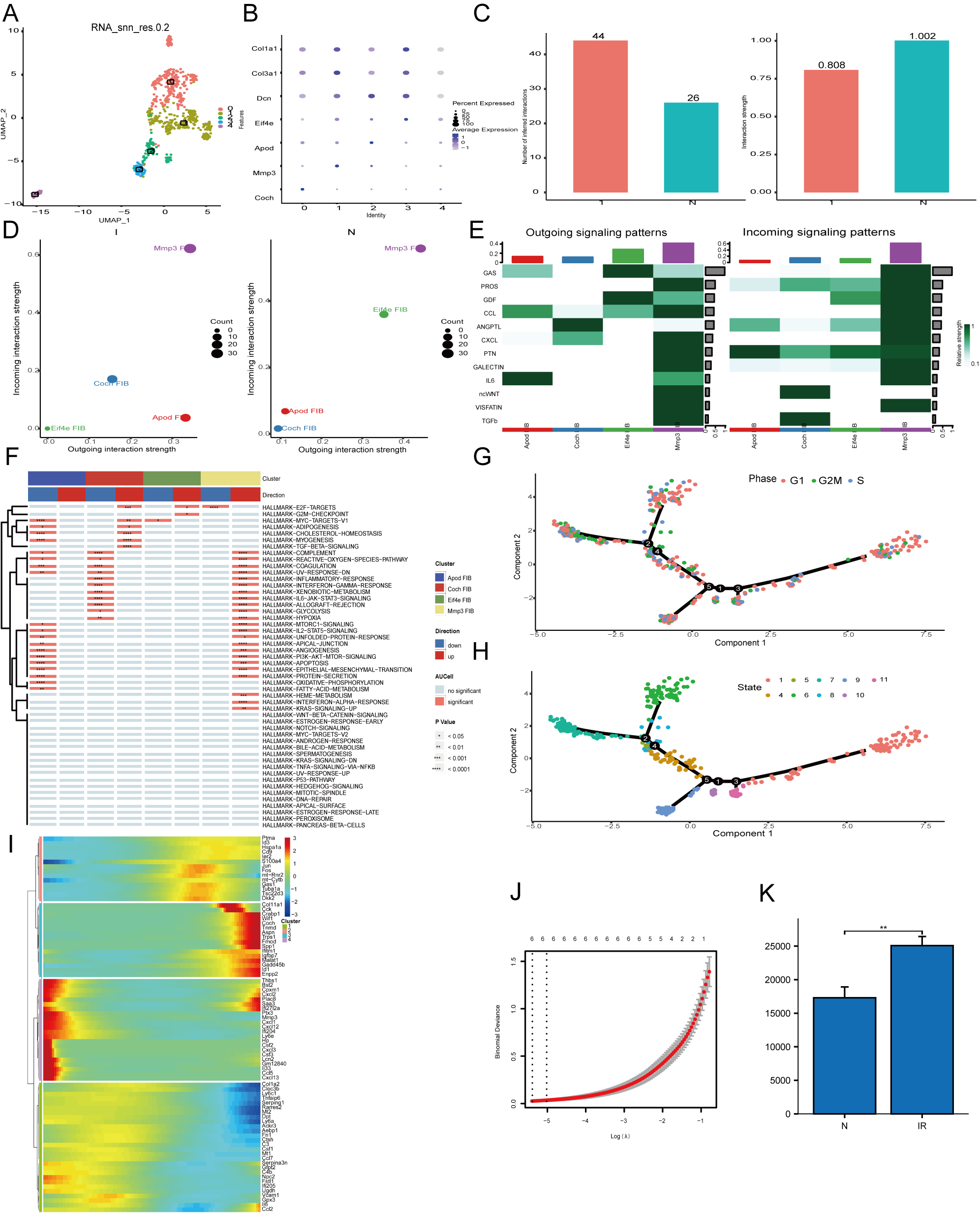


Supplementary Figure 2. (A) The UMAP map of fibroblasts at K = 0.2. (B) Characteristically expressed genes in different fibroblast subpopulations. (C) The bar plot shows the number and intensity of fibroblast signaling pathways in the irradiation and normal groups. (D) Scatter plot of the intensity of cell signaling communication of fibroblast subtypes in the irradiated and normal groups. (E) Outgoing and incoming signaling pathways of different fibroblast subtypes. (F) Heatmap showing the results of AUCell enrichment analysis of fibroblast subtypes. (G) UMAP plot showing the cell cycle of fibroblasts in the pseudotime trajectory. (H) UMAP plot showing a cluster of fibroblasts in the pseudotime trajectory. (I) Fibroblast differentiation differential gene heatmap. (J) Lasso machine-learning algorithm threshold selection. (K) The bar plot shows TGFBR2 gene expression in the irradiated and normal groups.

Supplementary Table 1. MMP3 Fibroblast subtype related genes(top50)

| MouseGene | HumanGene | log2FC | p.value.adj |
| --- | --- | --- | --- |
| Fn1 | FN1 | 0.784482545324377 | 8.89820669389146e-20 |
| Ctsh | CTSH | 0.876381255460368 | 2.20865368933966e-17 |
| Aebp1 | AEBP1 | 0.932292129049191 | 4.03317931964752e-17 |
| Ackr3 | ACKR3 | 0.987812492375329 | 2.28310502709568e-16 |
| Efemp1 | EFEMP1 | 0.886096584174649 | 2.70682588890545e-15 |
| Mmp3 | MMP3 | 1.77350872508061 | 2.88885860210622e-15 |
| Rarres2 | RARRES2 | 0.954910402974317 | 1.27220003492797e-14 |
| Tnxb | TNXB | 0.860566987143493 | 3.11379475554204e-14 |
| Csf1 | CSF1 | 1.22931081942627 | 5.16122183922452e-13 |
| Clec3b | CLEC3B | 0.593720533356347 | 7.15009735030533e-13 |
| Gda | GDA | 0.738350608578311 | 8.81857362697598e-13 |
| Cndp2 | CNDP2 | 0.717997317203975 | 2.77008080923875e-12 |
| Cd9 | CD9 | -0.840297539 | 9.09316972831797e-12 |
| Sulf2 | SULF2 | 0.660486852164024 | 6.95794821021076e-11 |
| Ccl2 | CCL13 | 1.35758035913259 | 8.21809362388007e-11 |
| Fstl1 | FSTL1 | 0.693081259814172 | 8.50879971298266e-11 |
| Creb5 | CREB5 | 0.699256363442084 | 1.00131654773878e-10 |
| Igfbp7 | IGFBP7 | -0.968504306 | 3.33610549074891e-10 |
| C3 | C3 | 1.35204645929562 | 5.51203797343065e-10 |
| Scara5 | SCARA5 | 0.536624545695757 | 6.84603686795381e-10 |
| Mgst1 | MGST1 | 0.642182936290199 | 7.52277691241873e-10 |
| Id3 | ID3 | -1.142607788 | 2.13655519785175e-09 |
| Ifi205 | IFI16 | 0.98947863652285 | 3.20282610416206e-09 |
| Ugp2 | UGP2 | 0.589215527930992 | 3.94488793900889e-09 |
| Ces2g | ANXA3 | 0.548186128064499 | 5.7428763222404e-09 |
| Gadd45b | GADD45B | -0.909688847 | 1.61288973018096e-08 |
| Tgfbr2 | TGFBR2 | 0.713633576320479 | 1.92623449430772e-08 |
| Twist1 | TWIST1 | -0.709382661 | 2.61160506720677e-08 |
| Sod3 | SOD3 | 0.7264114676456 | 4.94354447509877e-08 |
| Ndrg1 | NDRG1 | 0.967486023060882 | 6.07023594823041e-08 |
| Gas1 | GAS1 | -1.236203564 | 6.17886640518236e-08 |
| Grem1 | GREM1 | 1.34246102101753 | 6.596807972188e-08 |
| Igfbp6 | IGFBP6 | 0.736057257971585 | 8.78544941384048e-08 |
| Col1a2 | COL1A2 | 0.578754509834655 | 9.43711899044856e-08 |
| Thbs2 | THBS2 | 0.751822461747738 | 1.00535890365893e-07 |
| Ugdh | UGDH | 0.680708770251731 | 1.14227326769692e-07 |
| Jun | JUN | -1.054004416 | 1.70855364621767e-07 |
| Npc2 | NPC2 | 0.721419846445341 | 2.09209593316194e-07 |
| Casp4 | CASP4 | 0.611229776758097 | 3.04833157895532e-07 |
| Serpina3n | SERPINA3 | 1.21242354662463 | 3.88194007814965e-07 |
| Ifi204 | IFI16 | 0.663750813212151 | 6.6261343366015e-07 |
| Enpp2 | ENPP2 | -1.327740586 | 9.44610338674267e-07 |
| S100a16 | S100A16 | 0.751393134078598 | 1.23566125512864e-06 |
| Slit2 | SLIT2 | -1.171679493 | 1.49232054959693e-06 |
| Gpnmb | GPNMB | 0.968078932735661 | 1.96122444308422e-06 |
| Gpm6b | GPM6B | -0.57417175 | 2.43022255087828e-06 |
| Col3a1 | COL3A1 | 0.606210493611892 | 3.60897118278914e-06 |
| Slc43a3 | SLC43A3 | 0.664223315457053 | 5.72871318354554e-06 |
| Clk1 | CLK1 | -0.738574288 | 5.86971310011819e-06 |
| Ly6e | LY6E | 0.847057213726423 | 6.12144820931826e-06 |
